# Maximum soil organic carbon storage in Midwest U.S. cropping systems when crops are optimally nitrogen-fertilized

**DOI:** 10.1371/journal.pone.0172293

**Published:** 2017-03-01

**Authors:** Hanna J. Poffenbarger, Daniel W. Barker, Matthew J. Helmers, Fernando E. Miguez, Daniel C. Olk, John E. Sawyer, Johan Six, Michael J. Castellano

**Affiliations:** 1 Department of Agronomy, Iowa State University, Ames, Iowa, United States of America; 2 Department of Agricultural and Biosystems Engineering, Iowa State University, Ames, Iowa, United States of America; 3 National Laboratory for Agriculture and the Environment, United States Department of Agriculture—Agricultural Research Service, Ames, Iowa, United States of America; 4 Department of Environmental Systems Science, Swiss Federal Institute of Technology, ETH-Zurich, Zurich, Switzerland; Beijing Normal University, CHINA

## Abstract

Nitrogen fertilization is critical to optimize short-term crop yield, but its long-term effect on soil organic C (SOC) is uncertain. Here, we clarify the impact of N fertilization on SOC in typical maize-based (*Zea mays* L.) Midwest U.S. cropping systems by accounting for site-to-site variability in maize yield response to N fertilization. Within continuous maize and maize-soybean [*Glycine max* (L.) Merr.] systems at four Iowa locations, we evaluated changes in surface SOC over 14 to 16 years across a range of N fertilizer rates empirically determined to be insufficient, optimum, or excessive for maximum maize yield. Soil organic C balances were negative where no N was applied but neutral (maize-soybean) or positive (continuous maize) at the agronomic optimum N rate (AONR). For continuous maize, the rate of SOC storage increased with increasing N rate, reaching a maximum at the AONR and decreasing above the AONR. Greater SOC storage in the optimally fertilized continuous maize system than in the optimally fertilized maize-soybean system was attributed to greater crop residue production and greater SOC storage efficiency in the continuous maize system. Mean annual crop residue production at the AONR was 22% greater in the continuous maize system than in the maize-soybean system and the rate of SOC storage per unit residue C input was 58% greater in the monocrop system. Our results demonstrate that agronomic optimum N fertilization is critical to maintain or increase SOC of Midwest U.S. cropland.

## Introduction

Maize and soybean cropping occupies more than 70 million ha in the U.S., representing a large stock of intensively managed soil organic C (SOC). Maintenance of this SOC is essential for future food production because crop yields are positively associated with SOC in the long term [1]. Soil organic C can increase crop yield by enhancing soil water holding capacity and nutrient retention [2–4]. Furthermore, N bound to C in soil organic matter (SOM) is frequently the largest source of N for the crop and the largest sink of N fertilizer inputs in modern grain cropping systems [5,6]. Accordingly, SOC impacts both crop yield and N losses to the environment.

The quantity of C stored in a soil represents a balance between C inputs and outputs. Crop residue (biomass excluding harvested material) is the most important C input in most conventional cropping systems. And in maize-based cropping systems, N fertilization is perhaps the greatest management factor affecting crop residue inputs [7]. Nitrogen fertilization can increase maize residue production by 40 to 50% in a maize-soybean rotation [8,9] and by 50% to >100% in a continuous maize system [8–10].

Nevertheless, the overall effect of N fertilizer application on cropland SOC remains unresolved [9,11–14]. Nitrogen fertilization has been reported to increase, decrease, or have no effect on SOC [9–12,15]. Positive effects of N fertilization on SOC have been explained by increased crop growth leading to greater residue inputs to soil [15]. Negative effects of N fertilization on SOC have been explained by enhanced SOC mineralization when microbial decomposition is otherwise N-limited [16].

The relative importance of these processes in controlling SOC response to N fertilization likely depends on the response of crop growth to N fertilization. We hypothesize that when N inputs are below the rate that maximizes yield (agronomic optimum N rate; AONR), added N stimulates crop growth, which increases crop residue inputs to the soil and thereby increases SOC. However, when N inputs are above the AONR, added N imparts no change in crop residue production but increases residual inorganic N [17], which alleviates microbial N limitation and thereby enhances SOC mineralization [16]. Crop response to N fertilization is site-specific and regional N fertilization recommendations do not account for this variability [18]. Hence, the same recommended N rate applied at two different locations could favor SOC storage through enhanced crop growth at one location but favor SOC mineralization through increased residual inorganic N at the other location. This site-to-site variability in crop response to N fertilization may generate inconsistent conclusions about the effect of N fertilization on SOC.

In this study, we attempted to resolve the inconsistent effects of N fertilization on SOC by evaluating change in surface SOC across a range of N rates empirically determined to be insufficient, optimum, or excessive for maximum maize yield. We measured long-term SOC change in two dominant Midwest U.S. cropping systems (continuous maize and maize-soybean rotation) at four Iowa locations spanning a range of climates and soils that is representative of rainfed maize production in the Midwest U.S.

## Materials and methods

### Nitrogen fertilization experiments

**Fig 1:**
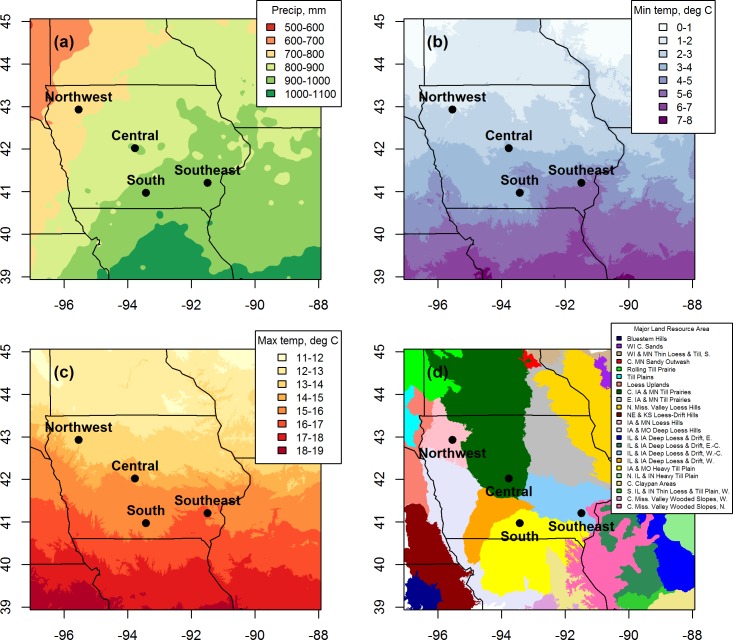
Locations of long-term N fertilization experiments. Maps show study locations within the most productive region of the U.S. rainfed Maize Belt (eastern Nebraska, southern Minnesota, Iowa, and central and northern Illinois) [20]. a) Mean annual precipitation, b) mean annual minimum temperature, c) mean annual maximum temperature, d) Major Land Resource Areas [21]. All climate data were averaged over 1981–2010 [22]. Cardinal directions for the Major Land Resource Areas are abbreviated in the legend (N, S, E, W, and C for north, south, east, west, and central, respectively).

Long-term N fertilization experiments were established at Iowa State University Research Farms to study the effect of N fertilization rate on crop yield and SOC storage in continuous maize and maize-soybean cropping systems. The experiments were established at the Central (42°01’ N; 93°47’ W), Southeast (41°11’ N; 91°29’ W), and South (40°58’ N; 93°25’ W) locations in 1999 and at the Northwest location (42°55’ N; 95°32’ W) in 2000 (Fig 1). The research sites span a climatic gradient from 790 to 1000 mm mean annual precipitation and 8.3 to 11.0°C mean annual temperature. Soils at all four locations are within the Mollisol order, and the suborders represent the three dominant suborders of the U.S. Maize Belt–Udolls, Aquolls and Ustolls [19]. The soil texture classes at the four locations are loam, silt loam, or silty clay loam and initial SOC concentrations ranged from 20.8 g kg^-1^ to 28.1 g kg^-1^ (Table 1). The Northwest, Central, and Southeast locations are underlain by artificial subsurface drainage, while the South location is not. All four research sites were previously managed as conventional maize and soybean production systems for at least 10 years before experimental establishment.

**Table 1:** Soil properties and weather conditions at four experimental locations in Iowa.

Location	USDA Soil Taxonomy	USDA Texture	Bulk density (g cm^-3^)	Initial soil organic C (g kg^-1^)	Initial soil total N (g kg^-1^)	Mean annual precipitation (mm)	Mean annual temperature (°C)
Northwest	Hapludoll-Haplustoll	Silty clay loam	1.11±0.02	27.9±0.2	2.45±0.02	790	8.3
Central	Hapludoll-Endoaquoll	Loam	1.36±0.03	20.8±0.6	1.69±0.04	970	9.0
Southeast	Argiudoll	Silty clay loam	1.08±0.02	28.1±0.2	2.19±0.02	1000	11.0
South	Argialboll-Argiudoll	Silt loam	1.27±0.03	22.4±0.1	1.75±0.01	980	9.5

Bulk density, initial soil organic C, and initial soil total N were measured on 0–15 cm soil samples and results are reported as mean ± SE. Mean annual precipitation and mean annual temperature were averaged over the study duration for each location (1999–2014 for Central and South, 2000–2014 for Northwest, and 1999–2015 for Southeast). Precipitation and temperature data are from Iowa Environmental Mesonet [23].

The experimental design at each location is a split-plot randomized complete block design with four replicate blocks. Each block consists of three main plots differing in crop sequence: continuous maize, maize-soybean, and soybean-maize. The main plots are split into individual treatment plots, each receiving one of five or seven N rates to maize; soybean does not receive N fertilizer. The N rates applied at the Central location are 0 to 269 kg N ha^-1^ in 67 kg N ha^-1^ increments, while those at the Northwest, Southeast, and South locations are 0 to 269 kg N ha^-1^ in 45 kg N ha^-1^ increments. Each individual treatment plot measures 6.1 m x 15.2 m (Northwest), 4.6 m x 15.2 m (Central and South) or 6.1 m x 19.8 m (Southeast). Crops are planted lengthwise in the plots with a row spacing of 0.76 m. The trials are managed with fall chisel plowing and spring secondary tillage before planting. Nitrogen fertilizer is applied as either urea incorporated at planting or urea ammonium nitrate solution injected as an early side-dress. Other nutrients and soil pH are maintained based on soil testing for optimum production.

### Yield measurement and residue carbon inputs

Maize and soybean grain yields were measured annually from the center three to six rows of each individual treatment plot using a small-plot combine. Mean grain yields were calculated by averaging across years (2000–2014 for Central and South locations, 2001–2014 for Northwest, and 2000–2015 for Southeast) by individual treatment plot. Maize and soybean grain yields are reported at standard 155 g kg^-1^ and 130 g kg^-1^ moisture contents, respectively. Mean grain yields were adjusted to dry matter and used to estimate total aboveground dry matter production using harvest indices of 0.50 grain dry matter/total aboveground dry matter for maize and 0.42 grain dry matter/total aboveground dry matter for soybean [24,25]. Aboveground residue inputs were calculated as the difference between total aboveground dry matter production and grain dry matter. Belowground residue inputs were calculated as the product of total aboveground dry matter production and a root/shoot ratio (0.18 root dry matter/total aboveground dry matter for maize and 0.15 root dry matter/total aboveground dry matter for soybean) [25]. Residue dry matter inputs were converted to residue C inputs assuming a tissue C concentration of 400 g kg^-1^ [26]. We estimated mean annual residue C inputs for each individual treatment plot by taking an average of maize and soybean total residue C inputs (aboveground plus belowground) weighted by the number of years in each crop phase.

### Soil sampling

Soil samples were collected at the onset of each experiment and again in 2014 (Northwest, Central, and South locations) or 2015 (Southeast location). For the initial sampling event, soil samples were collected by main plot at the Northwest and Central locations and by individual treatment plot at the Southeast and South locations. For the final sampling event, soil samples were collected by individual treatment plot at all locations. Each soil sample was composited from 15 cores of 2.5 cm diameter x 15 cm depth that were collected in the fall after harvest and before chisel-plow tillage. Soils were air dried and finely ground for total C and N determination by dry combustion elemental analysis using a Leco CN analyzer (initial sampling event; Leco Corp., St. Joseph, MI) or a Vario Max CN analyzer (final sampling event; Elementar Americas, Mt. Laurel, NJ). A subset of archived soil samples from the initial sampling event was also analyzed on the Vario Max CN analyzer to confirm consistency of results between instruments. Carbonates were not present in surface soil at these locations.

Because the plots at all locations are chisel-plowed annually to a depth of approximately 30 cm, the long-term N rate was not expected to influence bulk density in the surface soil. This assumption was corroborated at one location by previous research [10]. Accordingly, we used bulk density measurements taken from each cropping system within each block to scale SOC to a mass per area basis for the 0–15 cm depth. Rates of change in SOC and the C/N ratio were calculated for each individual treatment plot by first calculating the difference in surface SOC or C/N ratio between sampling times and then dividing this difference by the number of years between sampling times. Although historical sampling practices restricted our analysis of SOC to the surface 15 cm, changes observed in surface SOC may be representative of changes occurring at greater depths. An analysis of long-term SOC change data from regional agricultural studies showed that change in surface SOC was positively correlated with change in total soil profile SOC (S1 Fig).

### Data analysis

Quadratic-plateau regression models were fitted to mean maize grain yield and mean annual residue C input in response to N rate for each cropping system at each location using PROC NLIN (SAS ver. 9.4, SAS Inst., Cary, N.C.) [27]. In cases where no plateau was observed, quadratic regression models were fitted using PROC GLM (SAS ver. 9.4, SAS Inst., Cary, N.C.). Across-year means of grain yield and residue C input were not averaged across replicate plots prior to fitting regression models. We estimated the AONR for each cropping system at each location as the join point of the quadratic-plateau model fitted to maize grain yield. In cases where no plateau was observed, the AONR for maize grain yield was set to the highest N rate applied (269 kg ha^-1^) [5].

The EONR was calculated as the N rate at which the last increment of N input returns a grain yield increase large enough to pay for that input [18]. We calculated the EONR for each cropping system at each location using the maize grain yield response to N rate and assuming a price ratio of $0.0056 kg N^-1^/$1 Mg maize grain^-1^, according to grain prices for Iowa and N fertilizer prices for the North Central region between 1999 and 2015 [20]. Given the absence of a reliable soil test for determining appropriate N fertilizer applications, the EONR is among the most widely used tools to prescribe N fertilizer rates [28]. Seven Midwest land grant universities maintain an online tool that can be used to calculate the N rate that maximizes economic returns at a given fertilizer/grain price ratio for different regions based on multiple site-years (http://cnrc.agron.iastate.edu/). Because the online tool utilizes maize yield responses to N rate from many site-years (including new and discontinued trials not included in our study), the experimental site EONRs calculated for our study differ from the rates recommended by the online tool.

We used a quadratic regression model to represent the response of SOC change to N fertilizer rate (PROC MIXED; SAS ver. 9.4, SAS Inst, Cary, N.C.). For this analysis, we expressed N rate as a percentage of the location- and system-specific AONR (i.e., “relative N rate”). We used Akaike information criterion (AIC) to confirm that using the relative N rate as an explanatory variable produced a better fitting model than using the actual N rate. Crop sequence, crop sequence x relative N rate, and crop sequence x squared relative N rate were specified as fixed effects. Random effects included location, block nested within location, crop sequence x location, and crop sequence x block nested within location. Interactions of location and block with relative N rate did not account for additional variance when included as random effects. ESTIMATE statements were used to generate coefficient estimates for the maize-soybean cropping system, which included data from both the maize-soybean and soybean-maize sequences. The same approach was used to analyze change in the soil C/N ratio in response to relative N rate, except that a quadratic term for relative N rate was not included.

A linear regression model was used to represent the relationship between SOC change and mean annual residue C input (PROC MIXED; SAS ver. 9.4, SAS Inst, Cary, N.C.). Crop sequence and crop sequence x mean annual residue C input were specified as fixed effects. Random effects included location, block nested within location, crop sequence x location, and crop sequence x block nested within location. Because the range of mean annual residue C inputs differed between the two cropping systems (i.e., continuous maize had a wider range than maize-soybean), we tested for an effect of range on the relationship between SOC change and mean annual residue C input. Therefore, we constructed a data set that included SOC change data for the full range of residue C inputs provided by the continuous maize system and for the smaller range of residue C inputs provided by the maize-soybean system (i.e., “truncated range”). We then ran a model for the continuous maize system that included range (full vs. truncated), mean annual residue C input, and range x mean annual residue C input as fixed effects. Random effects included location and block nested within location.

Linear mixed-effects models were used to estimate least-squares means and standard errors or 95% confidence intervals for mean grain yield, mean annual residue C input, SOC change, and C/N ratio change at each N rate (PROC MIXED; SAS ver. 9.4, SAS Inst, Cary, N.C.). For mean grain yield and mean annual residue C input, the same subsets of data used for quadratic-plateau and quadratic regression model-fitting (i.e., each cropping system within each location) were analyzed with categorical N rate designated as the fixed effect and crop sequence nested within block as a random effect. For SOC change and C/N ratio change, the location x crop sequence x categorical N rate interaction was specified as the fixed effect. Random effects included block nested within location and crop sequence x block nested within location. LSMESTIMATE statements were used to generate least-squares means for the maize-soybean cropping system, which included data from both the maize-soybean and soybean-maize sequences.

For all statistics, we used alpha levels of 0.05 and 0.01 to indicate significant and highly significant results, respectively.

## Results

### Crop response to nitrogen fertilizer rate

**Fig 2:**
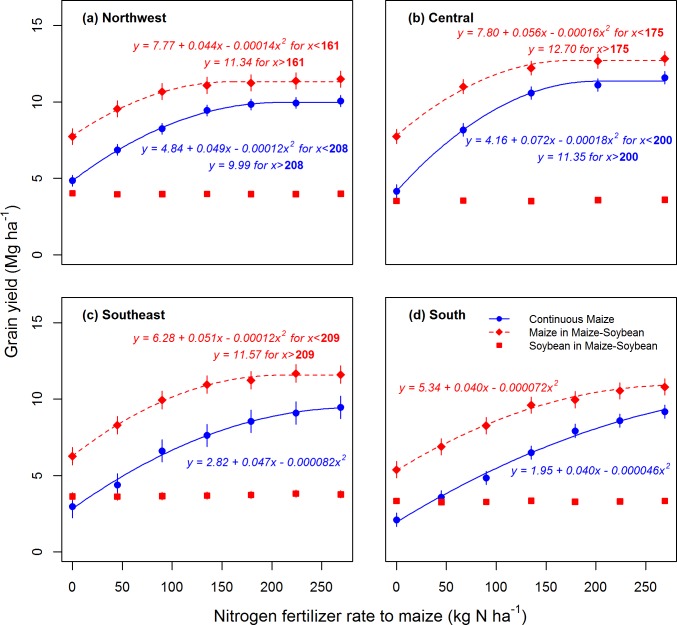
Cropping system and N fertilizer rate effects on grain yield. Mean maize and soybean grain yields in response to N fertilizer rate applied to maize in continuous maize and maize-soybean systems. Each of four Iowa study locations is shown on a separate panel (a-d). Grain yields were averaged across 14 (Northwest), 15 (Central and South), or 16 (Southeast) years according to the number of years between soil sampling events. Curves are quadratic-plateau or quadratic models fit to the data (*P* < 0.01 for all models). Bolded values are the agronomic optimum N rate (AONR) for quadratic-plateau curves. The AONR was set to the highest N rate applied (269 kg ha^-1^) for quadratic curves. Error bars represent 95% CIs, calculated using the variability in across-year mean grain yields among replicate plots. Confidence intervals for soybean yields are encompassed by the points.

For both cropping systems at all locations, maize grain yield increased with N rate, but the curvilinear responses indicate that yield gain per unit N applied decreased with increasing N rate (Fig 2). Maize yield was best fit by a quadratic-plateau model for the continuous maize system at two of the four locations (Northwest and Central; Fig 2A and 2B) and for the maize-soybean system at three of the four locations (Northwest, Central, Southeast; Fig 2A–2C). In cases where maize yield did not reach a plateau within the range of N rates applied (continuous maize at Southeast and South locations, maize-soybean at South location; Fig 2C and 2D), a quadratic model was used. The AONR ranged from 200 to 269 kg N ha^-1^ for the continuous maize system and from 161 to 269 kg N ha^-1^ for maize following soybean. Averaged across locations, the AONR was 16% greater for the continuous maize system than for maize following soybean.

**Fig 3:**
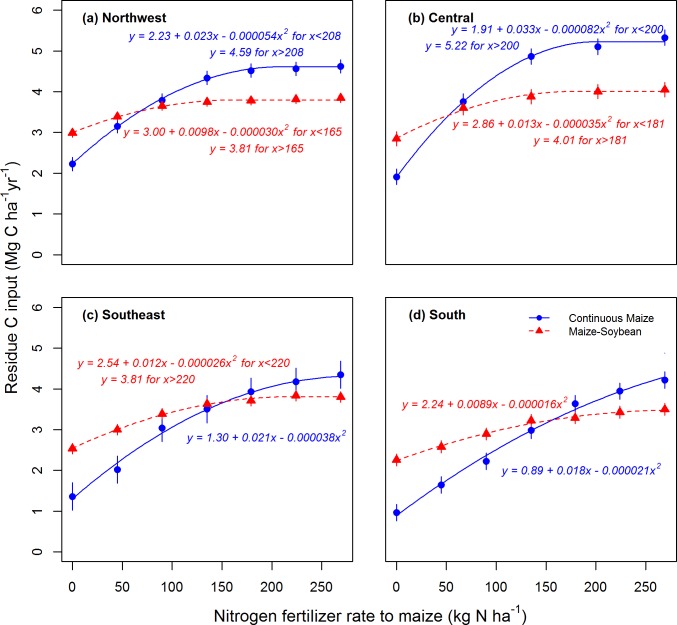
Cropping system and N fertilizer rate effects on mean annual residue C inputs. Estimated mean annual inputs of crop residue C in response to N fertilizer rate applied to maize in continuous maize and maize-soybean systems. Each of four Iowa study locations is shown on a separate panel (a-d). Curves are quadratic-plateau or quadratic models fit to the data (*P* < 0.01 for all models). Error bars represent 95% CIs, calculated using the variability in residue C inputs among replicate plots.

Averaged across locations, the difference between yield at the AONR and yield at the zero N rate was 6.6 Mg ha^-1^ for maize following maize and 4.8 Mg ha^-1^ for maize following soybean. Soybean grain yield was not affected by N rate applied to the previous maize crop at any location (Fig 2). Given these system differences in maize and soybean yield responses to N rate, it follows that the response to N rate of mean annual residue C input was greater for the continuous maize system than for the maize-soybean system (Fig 3). Averaged across locations, mean annual residue C input was 223% greater with optimum N application than with zero N application in continuous maize, but only 43% greater with optimum N application than with zero N application in maize-soybean (Fig 3). The optimally-fertilized continuous maize system produced 22% more residue C than the optimally fertilized maize-soybean system, averaged across locations.

### Soil organic carbon and nitrogen response to nitrogen fertilizer rate

**Fig 4:**
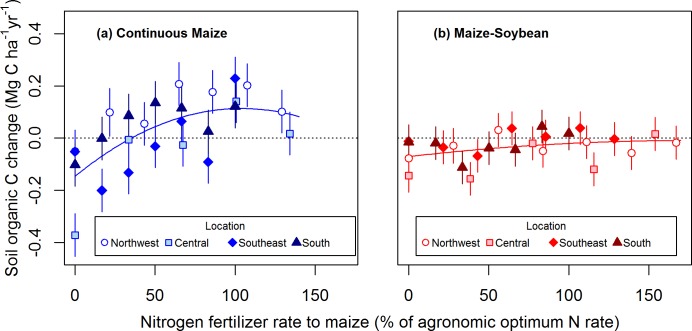
Cropping system and N fertilizer rate effects on soil organic C storage. Mean (± SE) annual change in surface (0–15 cm) soil organic C (SOC) in response to N fertilizer rate applied to maize in continuous maize (a) and maize-soybean (b) systems. Nitrogen fertilizer rate is expressed as a percentage of the agronomic optimum N rate for each system within each location. Quadratic regression curves are shown for both cropping systems, but the linear and quadratic coefficients were not significant for the maize-soybean rotation (continuous maize: y = -0.15 + 0.0050x – 0.000024x^2^, *P* < 0.01 for all coefficients; maize-soybean: y = -0.071 + 0.00070x – 0.0000020x^2^, *P* < 0.05 for intercept, *P* = 0.28 for linear coefficient, *P* = 0.66 for quadratic coefficient). For reference, the horizontal dotted lines represent no SOC change.

We evaluated SOC change over time in response to N fertilizer rate expressed as a percentage of the AONR. Scaling N rate to the location- and system-specific AONR substantially improved model fit (AIC of model with scaled variable = -159, AIC of model with original variable = -143). In the absence of N fertilizer inputs, SOC in the surface 15 cm declined by 0.15 Mg C ha^-1^ yr^-1^ in the continuous maize system (*P* < 0.01; Fig 4A) and by 0.07 Mg C ha^-1^ yr^-1^ in the maize-soybean system (*P* < 0.05; Fig 4B). There was no significant effect of cropping system on SOC change at the zero N rate (*P* = 0.23). For continuous maize, the response of SOC change to relative N rate was best represented by a positive quadratic model (*P* < 0.01 for linear and quadratic coefficients). The model estimated that SOC change reached a maximum of 0.11 Mg C ha^-1^ yr^-1^ at 104% of the AONR and then decreased slightly above this rate. The relative N rate that resulted in a neutral SOC balance was 36% of the AONR (Fig 4A). For the maize-soybean rotation, there was a slight positive but insignificant trend in SOC change with relative N rate (*P* = 0.28 and *P* = 0.66 for the linear and quadratic coefficients, respectively), causing SOC change to be approximately neutral at 100% of the AONR (-0.02 Mg C ha^-1^ yr^-1^, *P* = 0.48). Averaged across all relative N rates (including zero N), the SOC balance for the maize-soybean rotation was approximately neutral (-0.04 Mg C ha^-1^ yr^-1^, *P* = 0.17).

**Fig 5:**
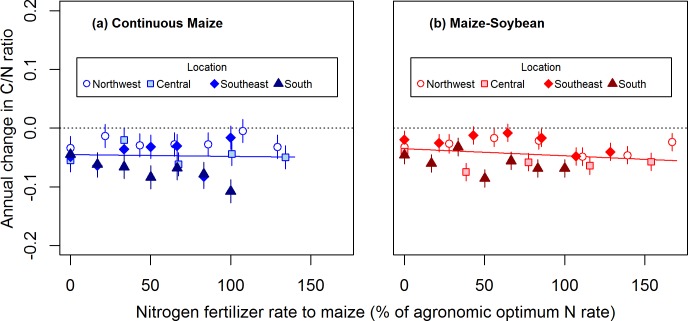
Cropping system and N fertilizer rate effects on change in the soil C/N ratio. Mean (± SE) annual change in the surface (0–15 cm) soil C/N ratio in response to N fertilizer rate applied to maize in continuous maize (a) and maize-soybean (b) systems at four Iowa locations. Nitrogen fertilizer rate is expressed as a percentage of the agronomic optimum N rate for each system within each location. Regression lines are shown for both cropping systems, but the slope was not significant for the continuous maize system (continuous maize: y = -0.045–0.00003x, *P* < 0.01 for intercept, *P* = 0.71 for linear coefficient; maize-soybean: y = -0.035–0.00012x, *P* < 0.05 for intercept and linear coefficient). For reference, the horizontal dotted lines represent no change in the C/N ratio.

Over 14 to 16 years of N applications, the soil C/N ratio decreased in both cropping systems (*P* < 0.05 for both y-intercepts; Fig 5). With zero N input, the C/N ratio declined by 0.045 units yr^-1^ in the continuous maize system and by 0.035 units yr^-1^ in the maize-soybean system. Change in the soil C/N ratio became more negative with increasing relative N rate in the maize-soybean system (*P* < 0.05) but not in the continuous maize system (*P* = 0.71).

### Soil organic carbon response to residue carbon input

**Fig 6:**
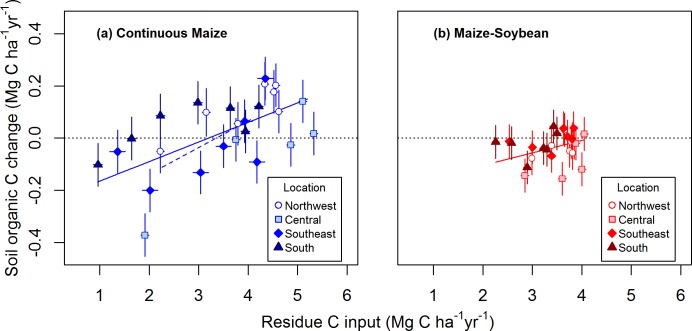
Relationship between soil organic C storage and residue C inputs. Mean (± SE) annual change in surface (0–15 cm) soil organic C (SOC) in response to mean (± SE) annual residue C inputs for continuous maize (a) and maize-soybean (b) systems. Solid regression lines apply to the full range of residue C inputs for each cropping system (continuous maize: y = -0.24 + 0.075x, *P* < 0.01 for both coefficients; maize-soybean: y = -0.20 + 0.048x, *P* < 0.05 for both coefficients). The dashed regression line for the continuous maize system was fitted using the range of residue C inputs equal to the range observed for the maize-soybean system; the regression lines for the full and truncated data ranges did not differ (*P* = 0.43). Some horizontal error bars are encompassed by the points. For reference, the horizontal dotted lines represent no SOC change.

There was a significant positive relationship between SOC change over time and mean annual residue C input for both cropping systems (*P* < 0.01 for continuous maize, *P* < 0.05 for maize-soybean; Fig 6). The SOC change was estimated to be -0.22 Mg C ha^-1^ yr^-1^ with zero residue C input for both systems (no cropping system effect on y-intercept, *P* = 0.81). However, the slope of the relationship between SOC change and residue C input was 58% greater for the continuous maize system than for the maize-soybean system (*P* < 0.01). This cropping system effect on the slope persisted when we performed the regression using a truncated range of residue C input values for the continuous maize system, which allowed us to use equal ranges of residue C inputs across the two cropping systems (*P* < 0.01; Fig 6). The residue C input level required to maintain SOC (i.e., the x-intercept) was 3.2 Mg C ha^-1^ yr^-1^ for the continuous maize system and 4.2 Mg C ha^-1^ yr^-1^ for the maize-soybean system.

## Discussion

### Agronomic optimum nitrogen fertilization benefits soil organic carbon storage

**Fig 7:**
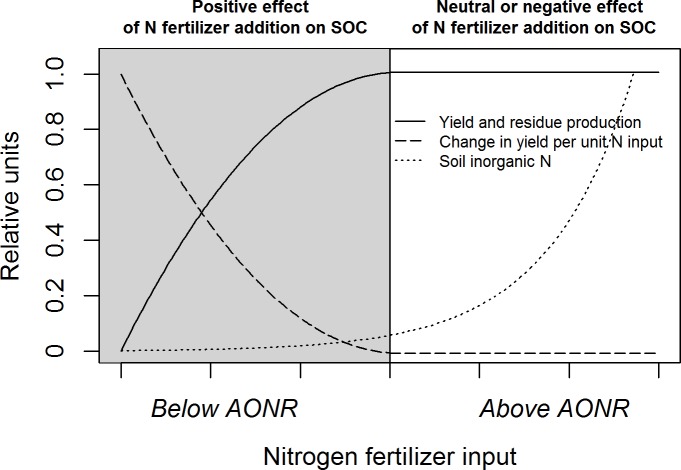
Conceptual relationships between N fertilizer input and maize yield, residue production, and residual soil inorganic N. The agronomic optimum N rate (AONR) is the N rate at which crop yield is maximized. Expected soil organic C (SOC) responses to fertilization of N-deficient maize (below the AONR, grey area) and N-sufficient maize (above the AONR, white area) are shown in bold above the plot.

Across a range of climates and soils that is representative of rainfed maize production in the Midwest U.S., N fertilization was necessary to maintain or increase SOC. Moreover, in the continuous maize system, the rate of SOC storage was maximized at approximately the same N rate that maximized maize yield and residue production. Our conceptual understanding of the SOC response to N fertilization is illustrated in Fig 7. When N inputs are below the AONR, added N stimulates crop growth, increasing crop residue inputs to the soil, thereby increasing the rate of SOC storage. When N inputs are above the AONR, added N imparts no change in crop residue production but increases residual inorganic N, enhancing SOC mineralization, thereby decreasing the rate of SOC storage. Residual soil inorganic N may enhance SOC mineralization by eliminating N limitation on microbial growth [12,16] or by decreasing soil aggregation [29,30], making previously protected SOM more susceptible to decay. Excessive N fertilization may also decrease the C/N ratio of maize residue [9], which can increase the decomposition rate of this organic matter input [31]. Multiple processes may operate to control the SOC response to N fertilization, but the importance of increased C inputs versus enhanced SOC mineralization depends on the N sufficiency level.

By accounting for site-to-site variability in the AONR, we resolve uncertainty regarding the response of SOC storage to N fertilization. Our findings suggest that studies comparing a sub-AONR to the AONR likely report a positive effect of added N on SOC due to enhanced crop residue production, whereas studies comparing a sub-AONR or the AONR to an excessive N rate may report neutral or negative effects of added N on SOC. Although our results demonstrate that N fertilization at the agronomic optimum rate is critical to maximize SOC storage of Midwest U.S. cropland, applying N at the AONR may not maximize sequestration of atmospheric C across the entire life cycle of N fertilizer. The positive effect N fertilization on SOC sequestration is typically partially offset by the CO_2_ released during fertilizer production, transportation, and application [32].

Interestingly, the surface soil C/N ratio decreased over time across all N rates in both cropping systems (Fig 5), suggesting an accumulation of total N relative to SOC. This shift may represent the fixation of ammonium by clay layers [33] or the replacement of high C/N ratio compounds with low C/N compounds during SOM turnover (e.g., through a shift in microbial community composition, and/or changes in soil aggregation) [34,35]. Soil organic matter with a low C/N ratio is typically more stable, with a longer mean residence time and relatively minor role in biological activity compared to SOM with a high C/N ratio [36].

### Crop productivity and carbon storage efficiency mediate soil organic carbon response to nitrogen fertilization

Our data highlight the importance of cropping system as a factor affecting the response of SOC to N fertilization and crop residue C input. When managed at the AONR, SOC increased over time in the continuous maize system, but did not change in the maize-soybean system. The greater response of SOC change to N fertilization for the continuous maize system was due in part to a greater N rate effect on residue C inputs for that system (Fig 3). With zero N application, the continuous maize system produced less residue than the maize-soybean system, leading to marginally greater SOC losses in the N-deprived continuous maize system than in the N-deprived maize-soybean system (Fig 4). On the other hand, the optimally-fertilized continuous maize system produced more residue than the optimally-fertilized maize-soybean system, leading to greater SOC storage in the optimally-fertilized monocrop system.

We were surprised to find that SOC change per unit residue C input was ~60% greater in the continuous maize system than in the maize-soybean system (75 vs. 48 kg SOC Mg^-1^ C input; Fig 6). As a result, the maize-soybean system required >30% more residue C input to maintain SOC stocks than the continuous maize system (4.2 vs 3.2 Mg residue C ha^-1^ yr^-1^). The transfer rates of residue C to SOC are within the range of those presented in a recent literature synthesis by Castellano et al. [37] and the inputs necessary to maintain SOC stocks are similar to or slightly higher than those reported by Kong et al. [38] for Mediterranean cropping systems (3.1 Mg C ha^-1^ yr^-1^) and Johnson et al. [26] for maize-based cropping systems (3.0 Mg C ha^-1^ yr^-1^). The lower proportion of crop residue C transferred to SOC in the maize-soybean system than in the continuous maize system may be caused by more favorable conditions for SOC mineralization in the crop rotation [39]. Whereas the continuous maize system returns relatively large quantities of residue to the soil every year, the alternating pattern of high and low residue inputs in the maize-soybean system may allow for more thorough decomposition of added C. The lower mass of the soybean residue than the maize residue likely keeps the soil warmer in the fall and spring [40] and the higher N concentration of soybean tissues may alleviate microbial N limitation that can occur during decomposition of maize residue [41].

### Management implications

Our results show that N fertilizer applications above or below the AONR may decrease the rate of SOC storage relative to agronomic optimum fertilization in maize-based cropping systems. Moreover, other research has shown that fertilizing above the AONR can increase nitrous oxide emissions [42] and nitrate leaching losses [17,43]. Although fertilizing at the AONR maximizes crop yield and SOC storage while avoiding major environmental N losses, this N rate may not necessarily minimize yield- or area-scaled N losses. Recent assessments of yield-scaled nitrous oxide emissions and nitrate leaching losses show that environmental risk per unit crop production is minimized when crop N uptake approximately balances the N fertilizer rate (i.e., neutral N surplus) [44,45]. Depending on site conditions, weather, and agronomic practices, fertilizing at the AONR may generate a positive N surplus and potentially substantial N losses [46].

Farmers seeking to maximize profit do not apply fertilizer at the AONR, but instead use the Economic Optimum Nitrogen Rate (EONR), which is the N rate that results in the maximum monetary return to N. The EONR is necessarily lower than the AONR because the incremental gain in grain yield per unit N fertilizer input declines with increasing N fertilizer inputs. We estimated the EONR to be 94% of the AONR for the continuous maize system and 88% of the AONR for the maize-soybean system for the locations in our study (S1 Table). Our estimates of SOC changes at the EONRs did not differ from those at the AONRs and were positive and neutral for the continuous maize and maize-soybean systems, respectively (S1 Table). In reality, the landscape of the Midwest U.S. comprises a mixture of continuous maize and maize-soybean production, with the maize-soybean rotation being the dominant system (between 1999 and 2015, maize was harvested on 52% of Iowa cropland, and soybean was harvested on 40% [20]). Maize grown in Iowa receives an average N fertilizer rate of 159 kg ha^-1^ [47], which is similar to the recommended rate to maximize economic returns (163 kg ha^-1^, average of recommended rates for continuous maize and maize-soybean systems, weighted by the area of each system; http://cnrc.agron.iastate.edu/). Considering that most Midwest U.S. cropland is in maize-soybean production and assuming that maize N application rates are close to the EONR, our study supports the idea that temperate cropland SOC is near steady-state [48,49].

## Conclusions

Our evaluation of N fertilization effects on SOC proved more meaningful, both statistically and conceptually, when we considered crop response to N fertilization. After scaling N rates to the location- and system-specific AONR, we found that applying N fertilizer at a rate optimal for maize growth was critical to maintaining SOC stocks in the maize-soybean system and maximizing SOC storage in the continuous maize system. The greater potential SOC storage in the optimally-fertilized continuous maize system than in the optimally-fertilized maize-soybean system was due to greater crop residue production as well as greater SOC storage efficiency in the monocrop system. For the continuous maize system, the rate of SOC storage increased with increasing N rate up to the AONR, but decreased above the AONR. This quadratic response suggests that the effect of N fertilization on SOC was mediated not only by increased crop residue production below the AONR, but also by increased SOC mineralization above the AONR. We conclude that multiple processes control SOC response to N fertilization and that the N sufficiency level can help explain their relative importance.

## Supporting information

S1 Fig Synthesis of long-term changes in soil organic C in topsoil vs. deeper soil. Data were compiled from studies of Upper Midwest U.S. Mollisols under agricultural management [11,50–54]. The dataset includes only chisel-plow tillage systems. The percentage change in soil organic C (SOC) for the topsoil was positively correlated with the percentage change in SOC for the total soil profile (y = -0.10 + 0.80x, r = 0.66). The slope of this relationship was not significantly different from one (*P* > 0.05). The relationship between percentage SOC change in topsoil vs. profile soil was evaluated using linear regression (PROC MIXED; SAS ver. 9.4, SAS Inst., Cary, N.C.). The model included random coefficients for each study assuming unstructured covariance to account for correlation among treatments within each study. A 95% CI was calculated for the slope of the line to determine whether it differed significantly from one.
(TIFF)

S1 Table Economic Optimum N Rates for each cropping system within each study location. Economic Optimum N Rates (EONRs) were calculated using yield response curves shown in Fig 2 and assuming a price ratio of $0.0056 kg N^-1^/$1 Mg maize grain^-1^. The estimated soil organic C changes at the EONRs for each cropping system were determined using regression curves shown in Fig 4.
(DOCX)

S2 Table Soil organic C and N concentrations and soil bulk density. Means and standard deviations of initial and final soil organic C and total N concentrations are presented for each N rate within each cropping system at the four study locations. Means and standard deviations of bulk density are provided for each cropping system at each study location.
(XLSX)
