# Supplementary figures and images for: Maximum soil organic carbon storage in Midwest U.S. cropping systems when crops are optimally nitrogen-fertilized
Source: PLoS One. 2017 Mar 1;12(3):e0172293. doi: 10.1371/journal.pone.0172293 (PMC5332021; doi:10.1371/journal.pone.0172293)

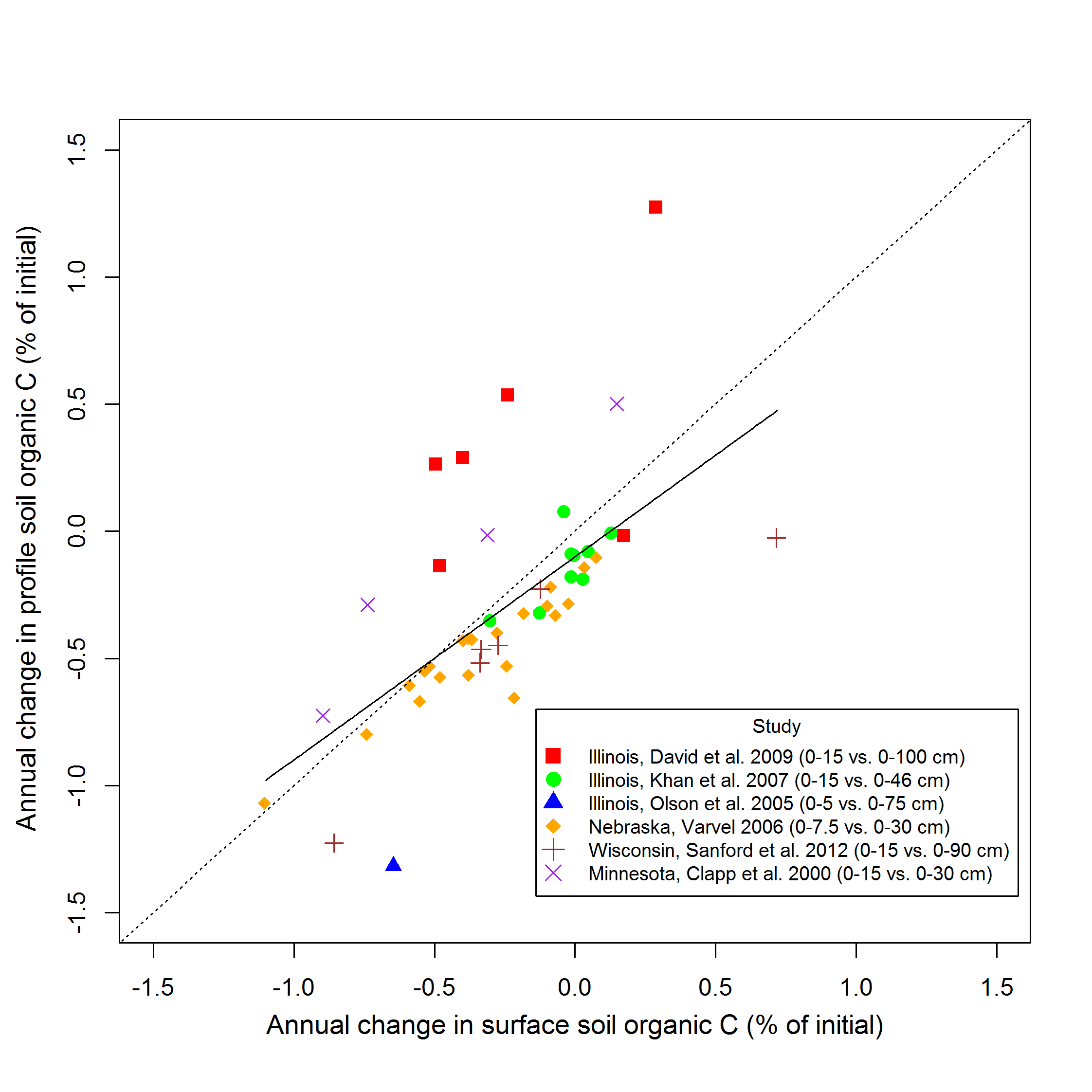

Supplement: S1 Fig — Data were compiled from studies of Upper Midwest U.S. Mollisols under agricultural management [11,50–54]. The dataset includes only chisel-plow tillage systems. The percentage change in soil organic C (SOC) for the topsoil was positively correlated with the percentage change in SOC for the total soil profile (y = -0.10 + 0.80x, r = 0.66). The slope of this relationship was not significantly different from one (P > 0.05). The relationship between percentage SOC change in topsoil vs. profile soil was evaluated using linear regression (PROC MIXED; SAS ver. 9.4, SAS Inst., Cary, N.C.). The model included random coefficients for each study assuming unstructured covariance to account for correlation among treatments within each study. A 95% CI was calculated for the slope of the line to determine whether it differed significantly from one. (TIFF) [file pone.0172293.s001.tiff]
