# Supplementary material for: Maximum soil organic carbon storage in Midwest U.S. cropping systems when crops are optimally nitrogen-fertilized
Source: PLoS One. 2017 Mar 1;12(3):e0172293. doi: 10.1371/journal.pone.0172293 (PMC5332021; doi:10.1371/journal.pone.0172293)
Supplement: S1 Table — Economic Optimum N Rates (EONRs) were calculated using yield response curves shown in Fig 2 and assuming a price ratio of $0.0056 kg N-1/$1 Mg maize grain-1. The estimated soil organic C changes at the EONRs for each cropping system were determined using regression curves shown in Fig 4. (DOCX) [file pone.0172293.s002.docx]

S1 Table.

| Location | System | EONR (kg N ha^-1^) |  |
| --- | --- | --- | --- |
| Northwest | Continuous Maize | 185 |  |
| Central | Continuous Maize | 185 |  |
| Southeast | Continuous Maize | 250 |  |
| South | Continuous Maize | 269 |  |
| Northwest | Maize-Soybean | 141 |  |
| Central | Maize-Soybean | 157 |  |
| Southeast | Maize-Soybean | 186 |  |
| South | Maize-Soybean | 236 |  |
| Location | System | EONR  (% of AONR) | Soil organic C change (Mg C ha^-1^ yr^-1^)* |
| Combined | Continuous Maize | 94 | 0.11 ± 0.03 |
| Combined | Maize-Soybean | 88 | -0.02 ± 0.03 |

*Mean ± SE. Soil organic C change was positive (*P* < 0.01) for continuous maize and neutral (*P* = 0.39) for maize-soybean.
